# Data describing the swelling behavior and cytocompatibility of biodegradable polyelectrolyte hydrogels incorporating poly(L-lysine) for applications in cartilage tissue engineering

**DOI:** 10.1016/j.dib.2016.02.077

**Published:** 2016-03-04

**Authors:** Johnny Lam, Elisa C. Clark, Eliza L.S. Fong, Esther J. Lee, Steven Lu, Yasuhiko Tabata, Antonios G. Mikos

**Affiliations:** a Department of Bioengineering, Rice University, Houston, TX, USA; b Department of Biomaterials, Institute of Frontier Medical Sciences, Kyoto University, Kyoto, Japan

**Keywords:** Hydrogel, Poly(L-lysine), Mesenchymal stem cells, Condensation, Chondrogenic differentiation, Cartilage tissue engineering

## Abstract

This data article presents data associated with the research article entitled “Evaluation of cell-laden polyelectrolyte hydrogels incorporating poly(L-lysine) for applications in cartilage tissue engineering” (Lam et al., 2016) [1]. Synthetic hydrogel composites fabricated using oligo(poly(ethylene glycol) fumarate) (OPF) macromers were utilized as vehicles for the incorporation of poly(L-lysine) (PLL) as well as the encapsulation of mesenchymal stem cells (MSCs). PLL-laden and PLL-free hydrogels were fabricated to characterize the main and interaction effects of OPF molecular weight, PLL molecular weight, and PLL loading density on the swelling and degradation of synthetic OPF hydrogels. Cells were then encapsulated within such hydrogels for in vitro culture and examined for viability, biochemical activity, and chondrogenic gene expression. These data, which are supplementary to the associated research article (Lam et al., 2016) [1], are presented here.

**Table:** 

**Specifications Table**	
Subject area	Polymer chemistry, tissue engineering, regenerative medicine, biology
More specific subject area	Hydrogel, biomaterials, mesenchymal stem cell, cartilage tissue engineering
Type of data	Table, figure
How data was acquired	Mass balance, microscopy
Data format	Figure is raw, table is analyzed
Experimental factors	Factorial study details in Table 1
Experimental features	Swelling ratio and degradation of hydrogels were derived from comparing the mass of swollen and dried hydrogels; main effects analysis was performed to identify both main and interaction effects of PLL loading parameters on hydrogel swelling and degradation; the LIVE/DEAD image was obtained from cell-laden, PLL-laden hydrogels
Data source location	Houston, Texas, USA
Data accessibility	Data is provided in the article

**Value of the data**

• Data will be informative for the design of synthetic hydrogels incorporating charged biomacromolecules (i.e. poly(L-lysine)) for stimulating phenotypic changes in co-encapsulated stem cells for applications in tissue engineering [1].
• Data from the full factorial analysis can help identify important fabrication parameters to modulate in order to control hydrogel swelling and degradation properties.

## 1 Data

**Fig. 1:**
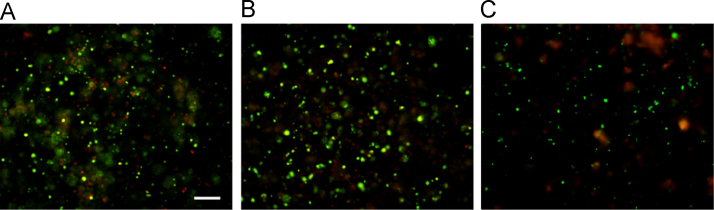
Viability staining of cells using LIVE/DEAD is shown for (A) non-PLL-laden controls and (B) PLL-laden hydrogels (500 ng/hydrogel) at 24 h and for (C) PLL-laden hydrogels (500 ng/hydrogel) at 7 days after cell encapsulation. Green: live cells, red: dead cells; scale bar = 200 µm.

The data provided here for the hydrogel formulations from the full factorial design (Table 1) represent the mean values for both the swelling ratio (Table 2) and the mass loss (%) (Table 3). Following these data are those describing the main and interaction effects of modulating hydrogel fabrication factors (Table 1) on the swelling and degradation properties of the resultant constructs. The cytocompatibility of the MSCs after encapsulation within PLL-laden OPF hydrogels is then shown in Fig. 1.

## 2 Experimental design, materials and methods

PLL-free 10 K and 35 K OPF hydrogels were utilized as negative controls. The nomenclature for the experimental groups is as follows: (OPF MW)(PLL MW)(PLL Dosage).

### 2.1 Swelling and degradation of PLL-laden OPF hydrogels

Swelling ratio (Table 2) and mass loss (Table 3) data, which were calculated by averaging the measured swelling ratio and mass loss (%) values for each formulation (*n*=4) at each time point over 28 days, respectively, were reported as the mean±standard deviation for samples at each time point. Following previously established procedures [2], [3], [4], fabricated hydrogels for the swelling and degradation study (*n*=4) were placed in 2 mL of PBS (pH 7.4) in a 24 well plate and incubated at 37 °C and under agitation (shaker table at 90–100 RPM) for 28 days. At days 1, 7, 14, and 28, the swelling ratio and mass loss of the hydrogels were determined using the following equations: swelling ratio = (*W*_s_– *W*_d_)/*W*_d_ and % mass loss=(*W*_i_–*W*_d_)/*W*_i_×100(%), where *W*_i_, *W*_s_, and *W*_d_ represent the weight of dried hydrogel immediately following fabrication before swelling, the weight of wet hydrogel after swelling at each time point, and the weight of dried hydrogel after swelling at each time point, respectively. Main and interaction effects were analyzed using a linear regression analysis methodology according to previously established methods [5], [6]. Differences observed in the main and interaction effects analysis were deemed significant if their standard error did not cross the zero line.

### 2.2 Factorial analysis of hydrogel swelling and degradation

The data presented in Table 4 were derived from the main effects analysis of the swelling and degradation of the various hydrogel formulations. A main effects and interactions analysis was performed using the SAS JMP Pro 11 statistical software package, as permitted by the factorial design, in order to formally examine the main effects of PLL MW and PLL loading amount on hydrogel swelling and degradation from the complex dataset over time according to established methods [5]. As shown in Table 4, the swelling of PLL-laden hydrogels was primarily affected by changes in the MW of the incorporated PLL, where increases in average PLL MW from 50 to 225 kDa resulted in increased swelling ratios at days 1 and 28. In addition to these main effects, several cross effects between the factors of PLL MW, PLL loading, and OPF MW were also observed at various time points. Namely, the swelling of hydrogels incorporating with PLL with an average MW of 225 kDa was more sensitive to changes in PLL loading amount and OPF MW. Indeed, changing the PLL loading amount from a low to a high level (AB interaction) and changing the OPF MW from a high to a low level (AC interaction) for hydrogels incorporating 225 kDa PLL both resulted in greater decreases in equilibrium swelling ratios when compared to formulations comprising 50 kDa PLL. Despite their effects on the swelling behavior of PLL-laden hydrogels, the factors of PLL MW and PLL loading amount nominally affected hydrogel degradation in general with the exception of several cross effects at day 7.

### 2.3 Assessment of cell viability encapsulated in OPF-PLL hydrogels

After characterizing the swelling and degradation of hydrogels, MSCs were then encapsulated into PLL-laden OPF hydrogels and assessed for viability using LIVE/DEAD staining and fluorescence microscopy. Cell viability was determined and verified at 24 h and 7 days after encapsulation using the Live/Dead staining assay (Life Technologies) following the manufacturer׳s instructions. Images (Fig. 1) were obtained using fluorescence microscopy (Nikon).

## Figures and Tables

**Table 1:** Full factorial study to characterize swelling behavior and degradation of PLL-laden hydrogels.

**Levels (*n*=4)**	**(A) PLL MW (kDa)**	**(B) PLL Dosage (per hydrogel)**	**(C) OPF MW (g/mol)**
Hi	225	20 µg	35 K
Lo	50	500 ng	10 K

**Table 2:** Mean swelling ratios of (a) 10 K and (b) 35 K OPF composite formulations.

**Formulation**	**PLL MW (kDa)**	**PLL Dosage (per hydrogel)**	**OPF MW (g/mol)**	**Mean swelling ratio**			
Day 1	Day 7	Day 14	Day 28
**a.)**							
10 K Control	––	––	10 K	15.2±0.7^b^	17.3±0.6^b^	14.4±1.4	20.3±0.6^a,b^
10 K 50Hi	50	20 µg	10 K	17.4±0.7^a^	17.8±0.4^a,b^	13.4±2.4	17.8±1.5^c^
10 K 50Lo	50	500 ng	10 K	16.7±1.3^a,b^	17.6±0.8^a,b^	14.2±1.4	20.7±0.4^a^
10 K 225Hi	225	20 µg	10 K	18.5±0.7^a^	16.9±0.6^b^	14.5±0.2	19.2±0.8^a,b,c^
10 K 225Lo	225	500 ng	10 K	17.7±0.9^a^	19.4±1.7^a^	12.4±1.8	18.4±1.2^b,c^
**b.)**							
35 K Control	––	––	35 K	16.3±0.6^c^	20.3±0.0^a^	20.3±0.2^a^	29.3±1.7^a^
35 K 50Hi	50	20 µg	35 K	18.9±1.3^a,b^	20.6±0.6^a^	20.7±1.0^a^	23.2±0.6^b,c^
35 K 50Lo	50	500 ng	35 K	17.7±1.7^b,c^	18.5±0.4^b^	20.1±0.6^a,b^	19.7±3.0^c^
35 K 225Hi	225	20 µg	35 K	20.9±0.2^a^	18.3±0.2^b^	18.4±0.6^b^	22.1±2.5^c^
35 K 225Lo	225	500 ng	35 K	21.0±0.9^a^	18.6±0.6^b^	20.4±1.2^a^	27.0±0.7^a,b^

For each time point, values not connected by the same letters (a, b, or c) are significantly different (*p*<0.05).

**Table 3:** Mean Mass Loss (%) of (a) 10 K and (b) 35 K OPF composite formulations.

**Formulation**	**PLL MW (kDa)**	**PLL Dosage(per hydrogel)**	**OPF MW(g/mol)**	**Mean mass loss (%)**			
Day 1	Day 7	Day 14	Day 28
**a.)**							
10 K Control	––	––	10 K	55.4±0.6^a,b^	59.0±1.8^a^	49.6±4.5	60.1±0.4
10 K 50Hi	50	20 µg	10 K	55.9±3.9^a^	62.0±0.7^a^	52.4±4.8	62.3±7.9
10 K 50Lo	50	500 ng	10 K	46.7±3.0^c^	51.4±2.7^b^	48.3±8.0	63.4±0.8
10 K 225Hi	225	20 µg	10 K	53.7±3.1^a,b^	51.2±2.3^b^	52.6±7.8	68.3±1.3
10 K 225Lo	225	500 ng	10 K	49.4±2.4^b,c^	58.5±4.3^a^	47.7±2.6	60.3±3.7
**b.)**							
35 K Control	––	––	35 K	46.5±3.3	59.2±1.4^a^	65.1±1.5	71.7±1.7
35 K 50Hi	50	20 µg	35 K	49.3±13.9	52.5±3.0^b^	63.0±5.9	68.5±1.9
35 K 50Lo	50	500 ng	35 K	45.0±4.3	56.1±4.8^a,b^	62.7±0.7	64.3±5.3
35 K 225Hi	225	20 µg	35 K	49.6±2.8	59.7±1.3^a^	61.7±4.9	66.9±6.4
35 K 225Lo	225	500 ng	35 K	52.1±6.4	55.0±2.4^a,b^	55.4±7.6	67.4±7.0

For each time point, values not connected by the same letters (a, b, or c) are significantly different (*p*<0.05).

**Table 4:** Main and cross effects on the swelling behavior and degradation of PLL-laden hydrogels.

**Swelling ratio**							
**Time**	**A**	**B**	**C**	**A×B**	**B×C**	**A×C**	**A×B×C**
Day 1	**0.917**±**0.366**	0.324±0.366	**1.010**±**0.366**	−0.164±0.366	−0.048±0.366	**0.415**±**0.366**	−0.172±0.366
Day 7	−0.183±0.284	−0.051±0.284	**0.550**±**0.284**	**−0.633**±**0.284**	**0.518**±**0.284**	**−0.386**±**0.284**	0.051±0.284
Day 14	−0.352±0.474	0.009±0.474	**3.139**±**0.474**	0.034±0.474	−0.351±0.474	−0.167±0.474	**−0.698±0.474**
Day 28	**0.653**±**0.565**	−0.430±0.565	**2.003**±**0.565**	**−0.583**±**0.565**	0.104±0.565	**0.889**±**0.565**	**−1.514**±**0.565**


**Mass loss**							
Day 1	0.010±0.011	0.019±0.011	−0.012±0.011	−0.015±0.011	−0.015±0.011	0.009±0.011	−0.002±0.011
Day 7	0.003±0.005	0.005±0.005	0.000±0.005	**−0.012**±**0.005**	−0.003±0.005	**0.012**±**0.005**	0.033±0.005
Day 14	−0.011±0.010	0.019±0.010	**0.052**±**0.010**	0.008±0.010	−0.003±0.010	0.010±0.010	0.007±0.010
Day 28	0.005±0.009	0.013±0.009	0.016±0.009	0.005±0.009	0.004±0.009	0.002±0.009	0.017±0.009

(A), (B), and (C) refer to the factors of PLL MW, PLL Dosage, and OPF MW as outlined in Table 1, respectively. Bold indicates significant main or cross effects (*p*<0.05).
