# Supplementary figures and images for: Data describing the swelling behavior and cytocompatibility of biodegradable polyelectrolyte hydrogels incorporating poly(L-lysine) for applications in cartilage tissue engineering
Source: Data Brief. 2016 Mar 4;7:614–9. doi: 10.1016/j.dib.2016.02.077 (PMC4802419; doi:10.1016/j.dib.2016.02.077)

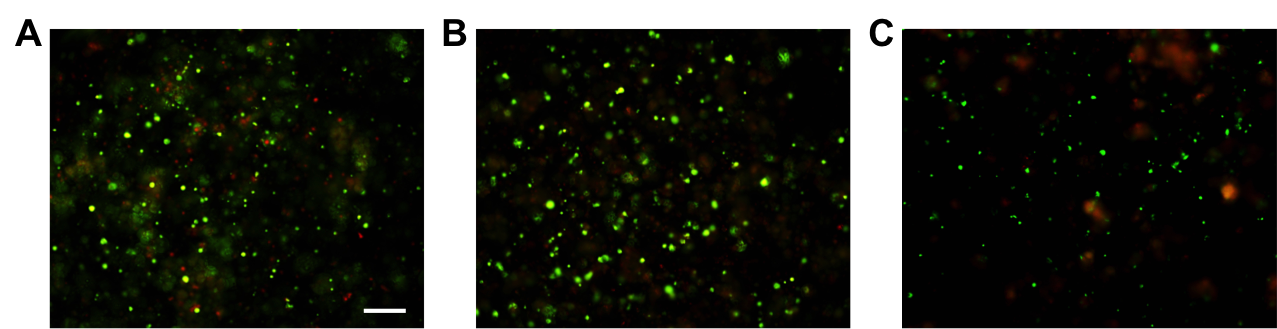

Supplement: Supplementary file 1 — Supplementary material [file mmc1.zip › New folder (2)/supplementalfigure1_v2.png]
